# From Single Level Analysis to Multi-Omics Integrative Approaches: A Powerful Strategy towards the Precision Oncology

**DOI:** 10.3390/ht7040033

**Published:** 2018-10-26

**Authors:** Maria Eugenia Gallo Cantafio, Katia Grillone, Daniele Caracciolo, Francesca Scionti, Mariamena Arbitrio, Vito Barbieri, Licia Pensabene, Pietro Hiram Guzzi, Maria Teresa Di Martino

**Affiliations:** 1 Department of Experimental and Clinical Medicine, Magna Graecia University, Salvatore Venuta University Campus, 88100 Catanzaro, Italy; mariaeugenia.gallocantafio@unicz.it (M.E.G.C.); k.grillone@unicz.it (K.G.); daniele.caracciolo1@studenti.unicz.it (D.C.); scionti@unicz.it (F.S.); 2 ISN-CNR, Roccelletta di Borgia, 88021 Catanzaro, Italy; mariamena.arbitrio@cnr.it; 3 Medical Oncology Unit, Mater Domini Hospital, Salvatore Venuta University Campus, 88100 Catanzaro, Italy; barbieri@unicz.it; 4 Department of Medical and Surgical Sciences Pediatric Unit, Magna Graecia University, 88100 Catanzaro, Italy; pensabene@unicz.it; 5 Department of Medical and Surgical Sciences, Magna Graecia University, 88100 Catanzaro, Italy; guzzi@unicz.it

**Keywords:** multi-omics data, biomarkers, precision medicine

## Abstract

Integration of multi-omics data from different molecular levels with clinical data, as well as epidemiologic risk factors, represents an accurate and promising methodology to understand the complexity of biological systems of human diseases, including cancer. By the extensive use of novel technologic platforms, a large number of multidimensional data can be derived from analysis of health and disease systems. Comprehensive analysis of multi-omics data in an integrated framework, which includes cumulative effects in the context of biological pathways, is therefore eagerly awaited. This strategy could allow the identification of pathway-addiction of cancer cells that may be amenable to therapeutic intervention. However, translation into clinical settings requires an optimized integration of omics data with clinical vision to fully exploit precision cancer medicine. We will discuss the available technical approach and more recent developments in the specific field.

## 1. Introduction

Omics technologies offer a new view of biological function and organization at level of different molecular systems. High-throughput studies generate genomic and transcriptomic data which finally lead to dissection of the cancer molecular profile. However, because human genomes are complex and regulated at multiple levels, a new challenge could be the integration of information coming from different biological layers. The integration of “omics” including genomics, epigenomics, transcriptomics, proteomics, and metabolomics, into physiological and clinical studies will provide new clues on the mechanism of tumor initiation, progression, and metastatic spread, as well as the discovering of novel targets for therapeutic intervention. Personalized medicine aims to ensure customized healthcare, which proposes disease prevention, medical decision, and tailored therapy for each condition/patient, taking information from integrative studies. However, omics integration is still in its infancy. Currently, single-omics analyses on cancer patient samples have provided valuable data available to the scientific community, mostly in the field of genomics, although matched clinical annotation is still limited. In this review, we first discuss general concepts on the generation of omics data, along with currently available strategies for multi-omics data analysis and integration in the context of clinical oncology. Finally, we discuss emerging challenges to translate information arising from this new knowledge into personalized anticancer therapies.

## 2. Omics Data Production

In the last decades, the improvement or development of new omics technologies notably ameliorated personalized medicine, in the prevention or treatment settings, by providing a broad range of information from genetic to metabolic tumor-specific features. Here, we will describe several exemplary studies reporting various applications of the main technologies, summarized in Table 1, adopted in cancer research to profile each biological layer.

### 2.1. Genomic Profile

The cancer genome carries several somatic changes that include single nucleotide substitutions, small insertions and deletions (indels), structural rearrangements, and copy number variations (CNVs). Some of these somatic alterations are targetable driver mutations, which contribute to cancer development and disease progression. The remaining ones are passengers, which have no “fitness” effects and thus do not contribute to cancer. However, the prevalence of somatic mutations shows inter- and intratumor heterogeneity ranging from approximately 0.001 per megabase (Mb) to more than 400 per Mb [1]. This variability is a consequence of clonal tumor evolution in which selection pressure in different contexts (microenvironment, host immune system, therapy) lead to a mosaic of different clones with varying degrees of somatic truncal and branched mutations in a Darwinian evolution process. In particular, tumors with increased genomic instability, such as melanomas, lung cancers, and cancers with DNA repair defects, are prone to develop branched aberrations [2]. Although many cancer drugs have been developed to specifically target clonal mutations in known truncal driver genes, which occurred early in cancer evolution, later branched abnormalities may confer resistance to therapies and thus may not account for the whole tumor evolution. The accurate detection of clonal and branched subclonal cancer mutations is a critical step in personalized cancer care as mutational profiling is used in clinical practice to classify patients in subgroups and associate the subtypes with clinical outcomes for better prognosis and treatment. McGranahan and colleagues identified approximately 15% of branched druggable mutations in *IDH1* in glioblastomas and approximately 20% in PI3K-AKT pathway effectors in all cancer types [3] Until now, a large amount of oncogenetic alterations from various tumor types have been collected in curated databases such as the Cancer Genome Atlas (TCGA), the International Cancer Genome Consortium, and the Cancer Cell Line Encyclopedia [4].

DNA sequencing technologies now allow targeted or whole exome sequencing (WES) and whole genome sequencing (WGS) of multiple tumors to identify genetic alterations. WES investigates the coding regions of the genome, whereas WGS focuses on the entire DNA sequence. In both, the tumor genome is compared with a patient’s germline sequence or a reference genome, thus parallel data capture and analysis is needed to classify variants as germline and somatic. Many reports demonstrate the power of massively parallel sequencing. For example, Wedge and colleagues [5] sequenced the whole genomes of 112 primary and metastatic prostate cancer samples. They identified 28 genes with an excess of coding driver mutations, five of which (*TBL1XR1*, *ZMYM3*, *IL6ST*, *CASZ1*, and *TBX3*) were previously unknown drivers in prostate cancer. They also reported loss of *CHD1* and *BRCA2* as early events in cancer development of ETS fusion-negative cancers and losses of *CDH12* and *ANTXR2* were associated with poorer recurrence-free survival. In addition, Miao and colleagues [6] analyzed the WES of 249 tumors and matched normal tissues from patients with clinically annotated outcomes to immune checkpoint therapy. They found that clonal driver alterations in *PIK3CA* and *KRAS* were enriched in patients with complete or partial response to treatment, while clonal driver mutations in *EGFR* were enriched in patients with progressive disease.

Complementary to WGS/WES, oligonucleotide array-based methods have been widely used to identify copy number (CN) changes as well as CN neutral differences associated with loss of heterozygosity (LOH). Currently, there are three types of chromosomal microarray analysis (CMA) platforms that differ in technology, resolution, and detection: array comparative genomic hybridization (array-CGH), single nucleotides polymorphism array (SNP-array), and array-CGH supplemented with SNP probes. Array-CGH platforms are designed for the detection of CN, while SNP-array and array-CGH plus SNP probes combine classic CN analysis with SNP genotyping. However, SNP density on CN+SNP platforms is typically lower than on the traditional SNP-array. For instance, Yeung et al. [7], in a retrospective analysis of 68 patients with myelodysplastic syndromes (MDS), found that 73% of patients had abnormal CMA, carrying either copy number LOH (32%) or CNV (41%). Patients harboring chromosomal abnormalities showed a lower overall survival (*p* = 0.04). A new and promising area of research is the use of liquid biopsies to genotype cell-free DNA (cfDNA) and circulating tumor cells (CTCs). Circulating cell-free DNA (cfDNA) is released from apoptotic or necrotic cancer cells. Circulating cell-free DNA harbors mutations, which are representative of the genetic background of the cell of origin and their levels are related to tumor stage and prognosis [8]. For example, Zill and colleagues [9] analyzed the somatic mutation landscape of 70 cancer genes from cfDNA deep-sequencing analysis of 21,807 patients with treated, late-stage cancers. They found that patterns and frequencies of driver alterations in advanced cancers reflect patterns found in early-stage disease. Circulating tumor cells derive from primary and metastatic tumor cells and are present into the bloodstream. Enumeration of CTCs has been proven as a prognostic marker for metastatic cancer and response to therapy [10]. Also, CTCs have similar clonal and subclonal structures with matched primary tumor. Comparative genomic analyses of CTCs, bone marrow (BM) tumor cells, and peripheral blood germline DNA from multiple myeloma (MM) patients showed that 100% of clonal mutations in patient BM were detected in CTCs and that 99% of clonal mutations in CTCs were present in BM MM [11]. Inherited germline variants were also associated with somatic mutations in known cancer genes, suggesting that a specific germline background may contribute to tumor development. Carter and colleagues [12] found that a germline haplotype at locus 19p13.3 increases likelihood of somatic mutations in *PTEN*. This locus includes two genes, *GNA11* and *STK11*, known to be involved in the PIK3CA/mTOR signaling pathway in which *PTEN* plays a major repressive role. In particular, GNA11 activates mTOR signaling whereas STK11 inhibits mTOR activity. The authors suggested a model in which the minor allele of 19p13.3 impairs mTOR signaling, conferring selective pressure to later somatic mutation of *PTEN*. In terms of cancer prevention, genome-wide association studies (GWAS) have identified many common low-penetrance germline variants that are linked with cancer predisposition, providing direct evidence of polygenic susceptibility. An example is the SNP rs149574 in *N*-acetyltransferase 2 (*NAT2*) gene, which is functional and has been reported to modify the effect of smoking in bladder cancer [13].

### 2.2. Epigenomic Profile

Epigenomic profiling provides the possibility to discover significant associations between chromatin features and genomic function at gene expression level. The epigenetic modifications, such as DNA methylation at the 5’ position of cytosine, or post-translational modifications of histone tails, are heritable changes that regulate gene expression pattern by altering DNA accessibility and chromatin structure without altering the genotype. As a consequence of these modifications, several biological processes are differentially modulated, sometimes producing pathologic conditions. In particular, defects in chromatin modifiers and remodelers have been associated with the etiology of both solid and hematological tumors [14]. Approximately thirty percent of all the reported cancer driver genes have been related to chromatin remodeling [15]. For example, alterations in epigenetic enzymes such as the Methylcytosine Dioxygenase (TET2) [16], the DNA Methyltransferase 3 α (DNMT3A) [17], and the Histone-Lysine *N*-Methyltransferase (EZH2) [18], as well as the aberrant promoter DNA methylation causing the silencing of genes such as *CDKN2A* or *MLH1* [19], have been associated with tumor development or progression. Considering the important role of the epigenetic enzymes in tumorigenesis, different targeted drugs have been developed. Among them, HDAC and DNMT inhibitors have been approved for treatment of hematological cancers and are under clinical evaluation for solid tumors and for combination protocols with non-epigenetic drugs, while many others drugs targeting other players in nucleosome remodeling are currently undergoing preclinical and clinical studies with very promising findings [20,21]. The idea to identify disease-related epigenetic biomarkers and to exploit the epigenetic reprogramming to revert a pathological state to a healthy one, led to deeper investigation of the human epigenome. For this purpose, next-generation technologies, combined with methylation microarrays, are being increasingly adopted to improve our understanding. In particular a strong interest is devoted to the DNA methylome whose characterization is essential to disclose cell-specific transcriptomes that could not be explained by limiting the analyses to a gene level. For DNA methylation profiling we can distinguish different genome-wide approaches such as (i) affinity enrichment-based methods [22,23], (ii) restriction rnzymes-based methods [24], (iii) bisulfite conversion-based methods [25,26], (iv) capture-based methods [27], and (v) third-generation sequencing based-methods [28,29]. All these techniques share the preservation of the methylation signature that would be otherwise lost during polymerase chain reaction (PCR) amplification needed for DNA sequencing. The readout of these approaches is entrusted to microarrays, such as the Infinium Human Methylation 450 Bead Chip array (Illumina, Inc., San Diego, CA, USA) that cover 450.000 CpGs, or sequencing [30]. Apart from the technologies adopted to reveal the DNA methylation profiling, it is worth mentioning other methods used to investigate the epigenome such as (i) chromatin immunoprecipitation sequencing (ChIP-Seq) [31,32], which is performed to identify chromatin-associated proteins; (ii) DNase I sequencing, assay for transposase-accessible chromatin sequencing (ATAC-Seq), and micrococcal nuclease sequencing (MNase-seq) [33,34] enabling to reveal chromatin accessibility; and (iii) chromosome conformation capture-on-chip sequencing (4C-seq) and high-throughput chromosome conformation capture sequencing (HiC-seq) which provide information about the global three-dimensional (3D) structure of the nucleus [35]. 

Many of the data produced through the technologies mentioned above are collected in several databases such as MethyCancer [36,37,38] including DNA methylation data; Histone Database [39] and HIstome [40] including histone modification data; and Cistrome cancer including chromatin remodelers data combined with tumor molecular profiling data. Several studies that focused on the epigenomic profiling of cancer patients led to the characterization of epigenetic players in tumor initiation or progression and to the identification of diagnostic or prognostic biomarkers. Guo and colleagues identified a signature of differentially methylated genes (*AGTR1*, *GALR1*, *SLC5A8*, *ZMYND10*, and *NTSR1*) as biomarkers for non-small cell lung cancer (NSCLC) diagnosis by integrating three high-throughput DNA methylation microarray datasets including 458 samples (352 NSCLC and 106 normal tissues) from GEO (Gene Expression Omnibus) and TCGA [41]. Exner et al. performed a microarray-based methylation analysis of 360 promoters of genes previously described as hypermethylated in several tumors on 22 rectal cancer DNA samples and eight control DNA samples identifying two novel three gene-based signatures comprising *TFPI2*-*DCC*-*PTGS2* and *TMEFF2*-*TWIST1*-*PITX2* which are able to label tumor samples respect to adjacent tissues or blood, respectively. Moreover, the authors found the methylation of *CDKN2A* as a negative prognostic factor for overall survival of rectal cancer patients [42]. Legendre and colleagues reported a differential methylation analysis performed through a paired-end whole-genome bisulfite sequencing on cell-free DNA from plasma of 40 metastatic breast cancer (MBC) patients that led to the identification of hypermethylation hotspots within CpG islands of 21 genes that are unique for MBC patients compared with a pool of 40 disease-free survivors or 40 healthy individuals. Their findings suggested that a DNA hypermethylation signature might be of prognostic relevance [43]. Qu and colleagues analyzed 111 Cutaneous T cell lymphomas (CTCLs) by ATAC-seq by identifying chromatin signatures which enable the discrimination between leukemic, host, and normal CD4^+^ T cells and by revealing that clinical response to HDAC inhibitors is associated with specific changes in chromatin accessibility. The regulome profiling could then be used as prognostic factor due to the patient-specific chromatin landscape in response to epigenetic drugs [44]. Cai and colleagues, through the 4C-capture method followed by next-generation sequencing, revealed genome-wide interacting partners in correspondence of the 8q24 locus by proposing an approach able to explain how genetic variants affect this well-known hotspot increasing prostate cancer risk [45].

### 2.3. Transcriptomic Profile

Transcriptomic technologies allow for the production of information on the total transcripts of a genome or a specific cell by the use of two high-throughput methods: (i) microarrays, which allow the simultaneous detection and quantification of thousands of previously identified transcripts by hybridization of targets on high-density array containing complementary probes; (ii) RNA sequencing (RNA-Seq), which uses high-throughput massive parallel sequencing combined with computational methods to detect and quantify the complete set of RNA transcripts. Comparison of transcriptomes in different tissues, conditions, time points, or even at single cell level gives information on how genes are regulated and differentially expressed disclosing details about the biology of the system. Moreover, expression profiles can also help to infer the functions of previously unannotated genes. Thereby, the lowering of the technology costs and increased sensitivity allowed a large amount of studies. Many consortium efforts have produced transcriptomic data sets of (i) cancer cell lines, such as the Encyclopedia of DNA Elements (ENCODE) [46], the Cancer Cell Line Encyclopedia (CCLE) [47], and Genentech [48]; (ii) normal tissues, such as the Genotype-Tissue Expression (GTEx) project [49] and the Human Protein Atlas (HPA) [50]; and (iii) tumor tissues such as TCGA [51] and the Stand Up To Cancer-Prostate Cancer Foundation (SU2C-PCF) project [52]. RNA-seq has become the most robust and comprehensive transcriptome profiling technology, virtually replacing all expression microarrays. An example of the clinical utility of RNA-seq has been demonstrated by several studies disclosing a large number of new actionable genetic events [53] or the real-time management of pediatric tumors [54] as well as the characterization of metastatic tumors [55]. Moreover, the advance in RNA-Seq library preparation methods, resulted in enhanced sensitivity and effectiveness of single-cell in situ RNA-Seq also performed in fixed tissues [56].

The applications of the transcriptome analysis span in a broad range of biomedical research, including diagnosis, disease classification, and monitoring, or response to, treatments. For example, Huet and colleagues [57] developed a predictor of progression-free survival based on a gene expression signature, including 23 genes, in follicular lymphoma. Using this strategy the authors were able to identify, at diagnosis, patients with an increased risk of progression when initially treated with rituximab and chemotherapy.

An additional example was reported by Boyault et al. who established one of the first transcriptomic molecular classification systems for hepatocellular carcinoma (HCC), composed of six groups, G1-G6 and G1-G3 groups were associated with a high rate of chromosomal instability and overexpression of proliferation genes. Specifically, the G3 subtype was reported as having the worst prognosis, bearing increased allelic loss, including chromosome 17 deletion and *TP53* mutation, *CDKN2A* hypermethylation, and increased expression of cyclins [58]. Further studies established other transcriptomic HCC signatures correlated with adverse biologic features and clinicopathologic observations [59]. Apart from gene expression profiling, transcriptomics has also been applied to non-coding RNAs (ncRNA), which are untranslated transcripts with several biological functions [60]. Many of the ncRNAs affect disease states, including cancer, cardiovascular, and neurological diseases. A class of ncRNAs, the microRNAs (miRNAs), have been widely analyzed by expression to generate miRNA signatures able to stratify human tumors in different subtypes correlating with clinical features or to be used as biomarkers or candidate therapeutic targets [61,62]. miRNA alterations were identified during breast cancer transition from ductal carcinoma in situ to invasive ductal carcinoma by Volinia and colleagues. A nine-microRNA signature was identified and specifically, let-7d, miR-210, and miR-221 were downregulated in the in situ and upregulated in the invasive transition, thus featuring an expression reversal along the cancer progression path [63].

Based on miRNA expression profiles, Namkung and colleagues identified three pancreatic ductal adenocarcinoma tumor subtypes associated with prognosis. These subtypes showed significantly different survival for patients with similar clinical features, demonstrating that the prognostic molecular subgroup has independent prognostic utility [64]. Li and colleagues reported novel diagnostic tools for deeper prognostic substratification in five intrinsic subtypes of primary glioblastoma (GBM) in TCGA dataset based upon miRNA expression profiles. miRNA signatures revealed that high-risk scores strongly correlated with poor overall survival as compared with patients who had low-risk scores. All these evidences suggest that transcriptomic technologies allow the identification of genes and pathways that can be associated with oncodriver signatures. Moreover, the nontargeted nature of transcriptomics lets us define novel transcriptional networks in complex systems such is the cancer disease.

### 2.4. Proteomic Profile

Proteomics is the study of the entire set of proteins in any given cell, including the set of all protein isoforms and modifications, the interactions between them, the structural description of proteins, and their higher-order complexes [65,66]*.* Therefore, proteomics is the next step to study biological systems because proteins are responsible for most cellular processes; their analysis would more accurately reflect cellular status to determine the mechanism that underlies disease initiation, progression, and dissemination [67,68]. To analyze the complex protein mixtures with higher sensitivity, the main technology presently in use is mass spectrometry (MS) [69], combined with liquid chromatography or matrix-assisted laser desorption ionization (MALDI-TOF/TOF) [68,70,71]. However, new methods were recently developed for quantitative proteomic such as isotope-coded affinity tag (ICAT) labeling, stable isotope labeling with amino acids in cell culture (SILAC), and isobaric tag for relative and absolute quantitation (iTRAQ) [72,73,74]. X-ray crystallography and nuclear magnetic resonance (NMR) spectroscopy are two major high-throughput techniques that provide 3D structure of proteins [75]. These technologies are adopted in cancer research to generate data on (i) differential protein expression levels, (ii) protein–gene expression correlation, (iii) differential protein expression comparisons between different cancer phenotypes and subtypes, and (iv) the associations of protein expression with survival prognosis in cancer patients. Proteomic data are collected in several databases including PRIDE (proteomics identification database) and Global Proteome Machine [76]. Other databases such as KEGG, IPA (Ingenuity Pathway Analysis), Pathway Knowledge Base Reactome, or BioCarta include comprehensive data regarding metabolism, signaling, and protein interactions [77,78,79]. Global proteomic profile is increasingly being carried out in both cancer cell lines and patient-derived samples to provide information that are useful for cancer type classification [80,81,82,83] and drug sensitivity/resistance prediction [84,85].

Tyanova and colleagues used quantitative proteomics to examine the functional difference between breast cancer subtypes, related to energy metabolism, clearly selecting four proteins (HER2, Grb7, FOXA1, and MLPH) which may represent novel potential therapeutic targets [80]. Moreover, in ovarian cancer, the application of proteomic analysis highlighted the differential expression of five proteins (serotransferrin, amyloid A1, hemopexin, C-reactive protein, and albumin) which improved the diagnostic performance of the model discriminating between benign and malignant tumors. The identification of these proteins shed light on the molecular signaling pathways that are associated with ovarian cancer development [84]. Similarly, by using an iTRAQ approach combined with high-resolution MS analysis, several proteins were found dysregulated during gastric cancer progression, demonstrating their potential use as specific biomarkers and/or therapeutic targets [73]. Moreover, by using MS-based proteomic analysis on 130 clinical breast cancer samples, Yanovich and colleagues demonstrated intertumor heterogeneity across three breast cancer subtypes and healthy tissues, identifying four proteomic clusters. One of them represents a novel luminal subtype, characterized by increased PI3K signaling, demonstrating the importance of deep proteomic analysis for clinical decision-making [86]. The application of a quantitative targeted proteomic approach was also applied to identify four candidate biomarkers of drug resistance (GRP75, APOA1, PRDX2, and ANXA) in ovarian cancer cell lines and patient biopsies, after carboplatin and paclitaxel treatments [87].

In the last decades, a promising new array application, named reverse phase protein array (RPPA), has been developed to measure either total or post-translationally modified proteins [88,89]. This technology allows the investigation of protein–protein interactions or biochemical reactions revealing information on the cellular processes driving tumor growth and response to treatments in cancer patients. An example of the application of RPPA has been reported by Masuda and colleagues, which demonstrated that the level of ribosomal protein S6 phosphorylated at serine residue 235/236 (p-RPS6 S235/236) was most significantly correlated with the resistance of HCC cells to sorafenib. The high expression of p-RPS6 S235/236 was confirmed immunohistochemically in HCC biopsies from patients who responded poorly to sorafenib, suggesting a novel biomarker for drug resistance [90]. In recent years, additional RPPA data generation is improving, for identification of cancer subtypes and targeted therapy through integration with other data platforms.

### 2.5. Metabolomic Profile

Another way to understand the amount of endogenous proteins within a biological system is to measure the metabolome—the endpoint of the omics cascade. Metabolites are small molecules interacting with proteins helping various biological functions. Detection of metabolites is carried out in cells, tissues, or biofluids by either NMR spectroscopy or MS [91]. Metabolomics have important potential in oncology, including the early detection and diagnosis of cancer and as both a predictive and pharmacodynamic marker of drug effect for therapeutic evaluation [92]. Moreover, metabolomics can provide a link between laboratory and clinics, particularly because metabolic and molecular imaging technologies enable a non-invasive discrimination of metabolic markers in vivo [93]. Despite this, the knowledge about metabolomics in clinical cancer research is poor. However, metabolomics allows for a complete global assessment of a cellular state, taking into account genetic regulation, altered kinetic activity of enzymes, and changes in metabolic reactions. Thus, compared with genomics or proteomics, metabolomics reflects changes in phenotype, and therefore, functions [91]. Metabolomic data obtained from NMR or MS methods were analyzed to provide information about metabolic profile of samples, including quantitation and association of putative biomarkers with respect to a particular characteristic or outcome, such as tumor grade or response to therapy. To accomplish this, bioinformatic databases were created to store metabolomic data of endogenous metabolites of biological samples simultaneously, such as the Human Metabolome Database, Genome Alberta, and Genome Canada [94]. An example of a metabolomics approach has been reported in several studies such as in the analysis of patients with acute myeloid leukemia (AML), in which significant changes in carbohydrate metabolism in blood were assessed [95]. Specifically, high levels of the well-known oncometabolite 2-hydroxyglutarate (a product of IDH1/IDH2 mutations) were detected in AML patients with poor prognosis, suggesting its potential role as a prognostic marker [96]. Other studies reported an association between fatty acids metabolites and carcinogenesis. Serum levels of unsaturated free fatty acids were revealed to be diagnostic indicators of early-stage colorectal cancer [97]. Similarly, amino acids also play important role in tumor development. As a matter of fact, cancer cell studies indicated serine and glycine metabolism as necessary resources for cancer cell metastatization and malignant potential [98]. Moreover, decreased citrate and elevated spermine levels were detected in prostatic fluid from men with prostate cancer, compared with noncancer patients, identifying new prostate cancer biomarkers for clinical diagnostic [99]. Branched-chain amino acids, including leucine, isoleucine, and valine, were found at high levels in blood from patients with human pancreatic adenocarcinoma compared with healthy controls [100].

Therefore, metabolomics data can contribute, with other omics, to an integrative approach to decipher cancer disease facilitating and accelerating the clinical practice.

## 3. Integrative Analysis Tools

As discussed before, the application of high-throughput technologies for the monitoring of almost all the key players within cells (DNA, RNA, ncRNAs, proteins, or metabolites) leads to the possibility to investigate cancer samples at different levels. The ultimate goal of these efforts is to discriminate among cancers samples to deliver precision and personalized treatments. From a computer science point of view, the key challenge to be faced is the integration of such heterogeneous data, since many different works have demonstrated that integromic analysis provides the opportunity to better understand molecular phenomena [101]. For instance, Hofree and colleagues developed a strategy to cluster patients integrating clinical data and functional relationships among a set of genes [102]. In parallel the integration of transcriptome analysis with proteomic data (protein–protein interactions) has been used in ovarian cancer [103]. More recently, Singh and colleagues introduced a novel approach analysis able to integrate even the mutation analysis [104]. Consequently, the need for the introduction of novel frameworks, algorithms, and tools for the integrated analysis arose. Here we focus on available tools for the integrated analysis to offer to the reader a synergistic point of view [105].

(a) dChip-GemiNi [106] is a web server able to integrate and to analyze miRNA and mRNA expression data. It is based on the analysis of time-series data, i.e., a set of temporally sorted observations, in which for each time there exist both an mRNA and a miRNA observation. dChip-GemiNi may be used through a web interface and it may be also downloaded for running it in a local environment. In dchip-GemiNi experimental data are compared with respect to an experimental model derived from publicly available data. This model has been built using a workflow composed of four steps. Initially, existing databases have been mined to derive known associations among miRNA–mRNA (e.g., TargetScan [107]) and transcription factor (TF) sites. Such associations have been integrated with data derived from expression databases. These two steps produce an initial network of associations among TF, miRNA, and mRNA. Then significant motifs have been mined and ranked. Each motif is a small subgraph representing the association among TF, miRNA, and mRNA.

(b) MAGIA [108] is a web server for the integrated analysis of mRNA, TFs, and miRNA. It includes miRNA–mRNA associations derived from eight different databases, and it also includes experimentally validated TF–miRNA interactions and TF–gene interactions. The user that would analyze its own data using MAGIA needs to upload into the web server experimental miRNA and gene/transcripts expression data. MAGIA is able to mine time series experiments in which for each sample there exists a pair miRNA/mRNA experiment (referred to as matched data), and two-class experiment (referred to as unmatched data). Results of MAGIA are small networks of association among miRNA, mRNA and TFs ranked by score.

(c) mirConnX [109] is based on a genome-wide approach, i.e., the associations are analyzed on a genome scale, but only referring to human and mouse data. The approach of analysis is based on the comparison of a first network, derived from data extracted from public databases, and a second one derived from experimental data provided by the user. It considers both miRNA–mRNA associations derived from many existing databases and associations among TF and genes. Experimental data provided by the user are mined to build a network obtained by analyzing all the possible pairwise interactions between TFs, miRNAs, and genes across the samples/replicates. Networks are finally merged into a single model using a simple weighted sum function (S) producing a novel network in which edges, which are found in both networks, have a higher weight.

(d) IntegraMiR [110] analyzes experimental data whose samples belong to two classes (e.g., healthy vs. disease). Initially, it searches and ranks differentially expressed miRNAs and mRNAs. Then it focuses on the functional comparison of transcripts by performing a gene set enrichment analysis (GSEA). Finally, the associations among miRNA and mRNA are derived on the basis of expression levels and biological consideration derived from GSEA. Finally, association among genes and TFs are derived from existing databases. Resulting networks of association are mined to reconstruct motifs (i.e., association among miRNA–mRNA and TF) considering only differentially expressed genes. These FFLs are then organized considering the kind of deregulation and ranked by using a statistical approach and visualized to the user.

(e) XCMS Online is a new platform available through an online web interface. It enables the analysis of metabolomics data by integrating both genomic and proteomic data, such as LC–MS data. It is based on a cloud infrastructure, therefore, it enables the sharing of data easily among collaborator [111]. Ruffalo and colleagues [112] developed a model for the integration of expression data, mutational data, and protein interaction networks. The model is based on the use of protein interaction networks to integrate experimental data into a single model and then to derive knowledge by mining this network. Authors used the model to analyze cancer related genes. They used public available data from TCGA to predict a gene association with cancer, shows improved predictive power in recovering cancer-related genes in known pathways.

(f) The MR4Cancer [113] is a web server to prioritize key genes involved in cancer controls, also known as master regulator (MR) genes. Authors extracted all the cancer specific regulator genes for 26 cancer types by analyzing TCGA data and then they extracted regulators that are not directly related to cancer from public databases. The list of these regulators has been used to build a reference model. User may upload into the web server its own experimental data (e.g., expression data) and MR4Cancer outputs ranked MRs by enrichment testing against the predefined.

(g) The Cancer Systems Biology Database (CancerSysDB) is a database for the analysis of cancer-related data that integrate multiple data types and multiple studies [114].

As regarding the integration of the epigenomic data together with RNA expression profiling and clinical data, we can mention databases such as:

(a) MENT, which is a database containing integrated data of DNA methylation and gene expression of normal and tumor tissues together with clinical data from GEO and TCGA [115].
(b) MethHC, which includes a systematic integration of DNA methylation and mRNA/microRNA expression data from human cancers [109].
(c) Wanderer, which is a web tool allowing user-friendly access to gene expression and DNA methylation data from TCGA [116].
(d) MethCNA, a database in which raw array data obtained by Infinium HumanMethylation450 bead chip and deposited in TCGA and GEO databases are collected and re-analyzed through a pipeline that includes multiple computational tools and resources for omics data integration. In this database DNA methylation and copy number alteration data refer to exactly the same genetic loci from the same DNA specimen, providing an important advantage respect than other databases that instead integrate data deriving from different patients and platforms [117].

## 4. Integrative Analysis Approaches in Cancer Research

Several studies focused on the integration of multi-omics data in order to provide a global view of the tumor landscape, to accurately stratify patients that may or not benefit from current treatment options, as well as identify new potential targets and new diagnostic and prognostic biomarkers, often by taking advantage of the access to wide databases, such as those previously mentioned, collecting data from thousands of patients.

Among these datasets, TCGA is the most extensive, and includes multi-omics data deposited from many centers involved in the TCGA research network, as well as patient’s clinical metadata prospectively collected. TCGA data currently refers to 33 cancer types from more than 11,000 patients that have been obtained through different high-throughput technologies such as DNA-seq, SNP-based platforms, array-based DNA methylation-seq, microRNA-seq, RNA-seq, and RPPA by providing a comprehensive view of the human cancer molecular bases. Each platform produces data that are informative about DNA mutational status, SNP, methylation, loss of heterozygosity (LOH), copy number variation, miRNA expression, gene expression, and protein expression.

An example of the TGCA-based multi-omics data integration is represented by the study published from the Cancer Genome Atlas Network on the breast cancer molecular landscape. This study analyzed different sets of breast tumors, through the six platforms mentioned above, to provide an extensive characterization enabling the identification of many subtype-specific alterations. As matter of fact, thanks to the multi-omics cross-integration analysis, it has been found that the four main breast cancer subtypes (luminal A, luminal B, basal-like, and HER2^+^) have specific genomic, proteomic, and clinical features. This study highlighted druggable targets for each group such as many players of PI3K and RAS-RAF-MEK pathways in basal-like tumors in which enhanced activity of the HIF1α/ARNT pathway was also found, suggesting the possibility to use bioreductive drugs or angiogenesis inhibitors in this orphan disease. In HER^+^ tumors, mutations of PIK3CA, PTEN, INPP4B, IK3R1, and within HER-family members were identified instead, while in luminal/ER^+^ subtypes, there were many mutated genes including p53 and RB1 [118]. Moreover, the huge amount of information included in TGCA offers the possibility to perform sophisticated analyses with an enhanced statistical power by integrating multi-omics data, not only from different patients with the same type of cancers, but also from different tumor types (TCGA Pan-Cancer Project). In fact, Weinstein and colleagues demonstrated that analysis of the molecular aberrations and their functional roles across tumor types could extend therapies effective in one cancer type to others with a similar genomic profile. Thus, the Pan Cancer TCGA data set provides a major opportunity to develop an integrated picture of commonalities, differences, and emergent themes across tumor species [119]. Multiplatform integrated analysis of different cancer types revealed molecular classification within and across tissues of origin [120,121].

An application of integromics includes the development of models that combine proteomic and genomic data with the aim to accurately predict patient survival. Interestingly, Zhu and colleagues generated RPPA data from tumors for which genomic, transcriptomic, and clinical features have been previously collected in the TCGA database, in order to provide the possibility to integrate multi-omics data from different layers to globally characterize these tumors [122].

Using the same cell line, Akbani and colleagues integrated RPPA together with genomic and transcriptomic data from TCGA to identify similarities and differences in pathways and network biology within and across tumor lineages, as well as biomarker and target discovery spanning multiple tumor lineages by providing a framework for determining the prognostic, predictive, and therapeutic relevance of the functional proteome [120].

Apart from TCGA-based analyses, several studies focused on the integration of different platforms to answer specific questions about various cancer types. Koplev and colleagues performed an integrative analysis on 726 pan-cancer cell lines profiled for gene expression, protein expression, and phosphorylation by explaining key regulators mechanisms involved in tumorigenesis. In particular, they revealed enrichment for HDAC inhibitors as inducers of epithelial–mesenchymal transition and kinase inhibitors as mesenchymal-to-epithelial transition (MET) promoters [123]. Snyder and colleagues instead performed an integrated analysis of multi-omics data generated through WES, RNA-seq, and T cell receptor sequencing from 29 patients with locally advanced or metastatic urothelial carcinoma which were treated with the checkpoint-blockade atezolizumab. With this approach, they evaluated the role of somatic, immune, and clinical patient-specific features in response to atezolizumab [124].

Mancikrnaova and colleagues focused on the study of the medullary thyroid carcinoma by combining DNA methylation data with mRNA/miRNA expression data. They identified specific genes involved in tumor progression that were negatively regulated through promoter methylation and indicated JAK/Stat pathway as potential target in RETM918T medullary thyroid carcinomas in enhancing the antitumor activity of RET inhibitor vandetanib [125]. Piccolo et al. summarized genomic data in tracking pathways to explain how germline, genetic, and epigenetic variations regulate gene expression changes in normal cells, in order to identify mechanisms that underlie breast cancer susceptibility [126]. Robles et al. found biomarkers to allow early detection and prognostic assessment of lung cancer by combining data regarding genomics (DNA methylation), transcriptomics (miRNA and mRNA expression), and metabolomics data (pro-inflammatory cytokines and metabolites from urine) in order to provide additional information for therapeutic tailoring [127]. With a similar approach, Li and colleagues combined genomic, transcriptomic, proteomic, and metabolomic data obtained by profiling three cell lines that were representative of HCC, each with a different metastatic potential in order to evaluate the influence of metabolism in metastatization process. They revealed 12 altered genes at different levels of specific pathways involved in cell metabolism such as sucrose and glutathione metabolism and glycolysis. Moreover, they reported an association between uridinediphosphate (UDP)-glucose pyrophosphorylase 2 (UGP2) and cell migration and invasion in vitro and in vivo [128]. Recently, our group used an integromics approach to shed new light within the molecular architecture of multiple myeloma (MM) hyperdiploid (HD-MM) and the non-hyperdiploid (nHD-MM) subtypes. By integrating annotated MM patient mRNA/miRNA dataset information, a specific gene and miRNA expression profile for HD-MM was found. Indeed, from this analysis a significant role of the STAT3 pathway, as well as the Transforming Growth Factor β (TGFβ) and the transcription regulator nuclear protein-1 (NUPR1), was demonstrated, thus defining novel molecular features of HD-MM that may translate in novel relevant therapeutic targets characterization [129].

## 5. Discussion

**Figure 1:**
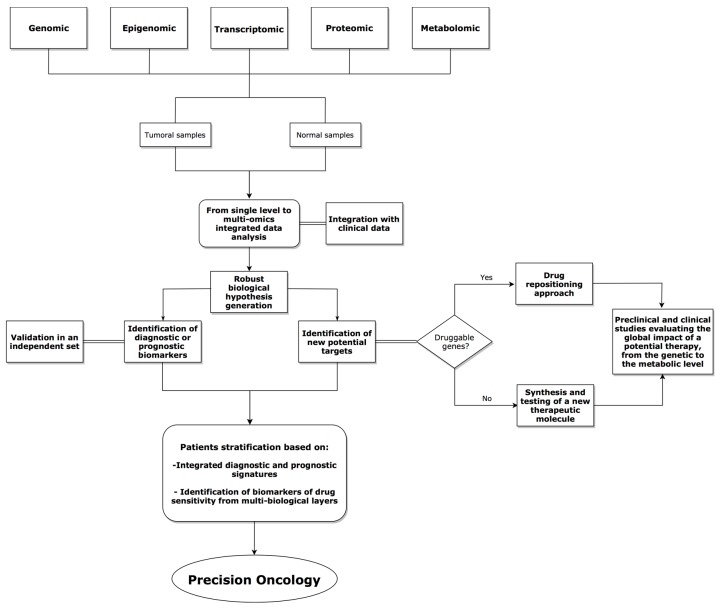
Representative flowchart of a multi-omics integrative-based approach for precision oncology.

Molecular approaches, including mutational analysis, RNA and miRNA expression profiling, and epigenetic characterization, greatly enhance the understanding of pathogenesis and allows prognostic stratification for many cancers, thus driving the rational design of novel targeted therapies. In addition, to gain a broader perspective of the molecular aberrations that contribute to tumor development, it is valuable to consider data at the pathway more than at single gene level. Furthermore, by tracking pathway activities using multiple types of omics data, including both genomic alterations and gene expression changes, it is possible to delineate a landscape view, which might include tumor cells and the micro-environment, providing a novel approach to define personalized therapeutics. Accordingly, the expression of cDNAs harboring mutations identified in human cancer if combined with protein functional assays may address whether these mutations are crucial for disease progression (driver mutations) or have been generated as the consequence of genomic instability, without biologic relevance (passenger mutations). High-throughput data may provide inputs for preclinical and clinical studies towards the precision medicine starting from the exploration of a wide amount of patient-specific omics information (see a possible work-flow represented in Figure 1).

Even if the theoretical value of precision medicine approaches as integrative genomics is now well-established, it has to be demonstrated its value in providing a paradigm shift in the real life. This is a crucial point that needs to be approached in terms of feasibility, effectiveness, and equity. It is clear that a major point is that all validated approaches must be offered in light of clinical utility, which means they provide useful information for clinical decision-making and not provide only redundant data. In this light, a biomarker-driven approach can allow patient selection and, therefore, reduce toxicity and costs, with a major benefit for patients, the health system, and other stakeholders. In terms of equity the major point is not indeed the cost of molecular analysis, which is lowering but a true access for all patients and a full access to all molecular targeted drugs which might be identified, that can be highly expensive. At this aim is crucial the setting up of laboratory networks with free sharing of molecular data and programs to negotiate access to innovative drugs for nationwide coverage. These considerations resound more for those patients with a rare disease, in which more specific and personalized treatments are eagerly awaited. In cancer treatment, precision medicine offers the opportunity to choose the drugs not only based on the type of cancer, but also on the genomic driver aberrations that can be tissue-agnostic but shared by different malignancies In drug development, precision medicine means finding new drugs that act as “keys” to certain “locks” in the body. In the case of rare disease, molecular diagnosis opens the door for new treatment options that cannot be explored by conventional pivotal trials.

All together these points show that the empowering of integrative genomics as a novel tool for precision medicine indeed is not only a technical but also a social and health policy challenge.

**Figure 2:**
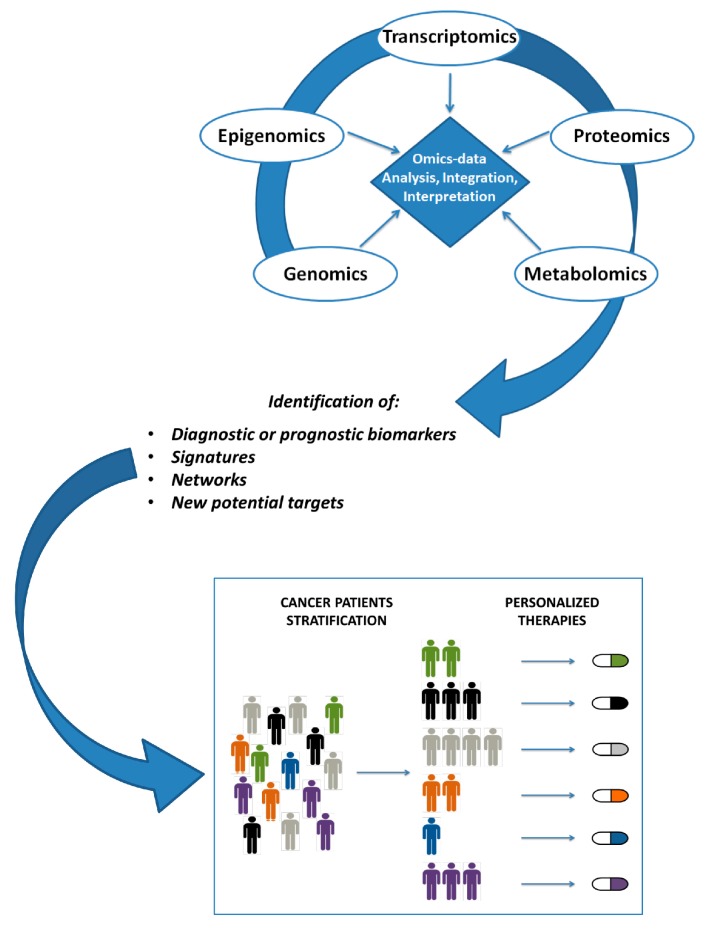
Schematic workflow summarizing major steps occurring from omics data production to personalized clinical decision-making.

The integration of omics profiles with clinical variables lead to improved prognostic performance over the use of clinical variables alone. In fact, the integrated portrait of omics architecture provides a comprehensive view which likely outperforms the predictive capability of single gene changes. Developing models that accurately can predict patient survival using prognostic and predictive biomarkers obtained from aggregation of multidimensional omics data, is a challenge in the era of precision oncology. A promising new direction for enhancing all the omics techniques is the integration of data-driven network models with prior biological knowledge. This strategy lead to a significantly improved biomarkers identification compared for example to the top genes obtained by conventional differential gene expression analysis. A great step forward in precision medicine could derive from a pan-cancer analysis of multiple omics profiles on a genome-wide scale, in order to understand the shared patterns across cancer types and identify shared actionable targets at a multilayer level. It therefore appears evident that the integration of omics data represents a powerful tool to allow clinical translation of this integrated dissection of cancer biology (Figure 2).

However, major efforts will be also necessary for the adoption in the clinics of raw data obtained from the described technology platforms, in order to translate wet biologic information to an improved therapeutic approach for cancer patients.

## Figures and Tables

**Table 1:** Summary of the technologies which have provided relevant information in cancer research to investigate different aspects of each biological level and are described in this article.

Data Type	Main Platforms		Applications
Genomic	Microarray	Array-CGH	Identification of CNVs
		SNP-array Array-CGH + SNP	Identification of CNVs, copy neutral of LOH, SNPs genotyping in defined sequences
	DNA-seq	WES	Identification of DNA mutations and CNVs
		WGS	
		Targeted exon-seq	
Epigenomic	Affinity enrichment-based methods	MeDip-Seq	DNA-methylation profiling
		MBD-Seq	
	Bisulfite conversion-based methods	BS-Seq	
		OxBS-Seq	
	Capture-based methods		
	Restriction enzymes-based methods		
	ChIP-Seq		Identification of chromatin-associated proteins
	MNase-Seq		Investigation of chromatin accessibility
	ATAC-Seq		
	DNase Il-Sseq		
	4C-Seq		Investigation of the 3D structure of the genome
	HiC-Seq		
Transcriptomic	Microarray		Quantification of a wide set of defined sequences simultaneously
	RNA-Seq		Detection and quantification of theoretically all RNA sequences including lncRNAs and microRNAs
Proteomic	LC–MS/MS		Analysis of complex protein mixtures with high sensitivity
	MALDI-TOF/TOF MS		
	ICAT		Labeled proteins quantification
	SILAC		
	iTRAQ		
	X-ray crystallography		Identification of the 3D structure of proteins
	NMR		
	RPPA		Quantification of either total proteins or post-translationally modified proteins
Metabolomic	NMR		Discrimination of metabolic markers
	MS		Analysis of complex metabolite mixtures with high sensitivity

CGH: Comparative genomic hybridization; CNV: copy number variation; SNP: single-nucleotide polymorphism; LOH: loss of heterozygosity; WES: whole exome sequencing; WGS: whole genome sequencing; MeDip: methylated DNA immunoprecipitation; MBD: methyl-CpG-binding domain; BS: bisulfite; OxBS: oxidative bisulfite; ChIP: chromatin immunoprecipitation; MNase: micrococcal nuclease; ATAC: assay for transposase-accessible chromatin; 4C: chromosome conformation capture-on-chip; HiC: high-throughput chromosome conformation capture; LC–MS/MS: liquid chromatography-tandem mass spectrometry; MALDI-TOF/TOF MS: matrix assisted laser desorption ionization time-of-flight; ICAT: isotope-coded affinity tag; SILAC: stable isotope labeling by/with amino acids in cell culture; iTRAQ: isobaric tags for relative and absolute quantitation; NMR: nuclear magnetic resonance; RPPA: reverse phase protein array; lncRNA: long non-coding RNA.
